# Factors Affecting Breast Myopathies in Broiler Chickens and Quality of Defective Meat: A Meta-Analysis

**DOI:** 10.3389/fphys.2022.933235

**Published:** 2022-07-01

**Authors:** Francesco Bordignon, Gerolamo Xiccato, Marija Boskovic Cabrol, Marco Birolo, Angela Trocino

**Affiliations:** ^1^ Department of Agronomy, Food, Natural Resources, Animal and Environment (DAFNAE), University of Padova, Padova, Italy; ^2^ Department of Food Hygiene and Technology, Faculty of Veterinary Medicine, University of Belgrade, Belgrade, Serbia; ^3^ Department of Comparative Biomedicine and Food Science (BCA), University of Padova, Padova, Italy

**Keywords:** white striping, wooden breast, spaghetti meat, risk factor, sex, breast yield, pH, cooking losses

## Abstract

Fast-growing broiler chickens are subjected to breast myopathies such as white striping (WS), wooden breast (WB), and spaghetti meat (SM). Available studies about risk factors for myopathy occurrence often used flock data whereas a few reports evaluated chicken individual data. Thus, the present study aimed to elucidate the effect of growth and slaughter traits, besides sex and genotype on myopathy occurrence. Data were obtained from eight experimental trials, which used a total of 6,036 broiler chickens. Sex, genotype, daily weight gain, slaughter weight, and breast yield were evaluated as potential risk factors by logistic regression analyses. Then, the effects of myopathy and sex were evaluated on meat rheological traits (pH, colour, cooking losses and shear force). Based on a logistic regression, WS occurrence was associated with genotype, breast weight, and breast yield. Compared with chickens with intermediate breast weight and breast yield, higher odds of having WS were found in chickens with high breast weight (OR: 1.49) and yield (OR: 1.27), whereas lower odds were found in those with low breast weight (OR: 0.57) and yield (OR: 0.82). As for WB and SM, females had lower odds of having WB (OR: 0.55) and higher odds of showing SM (OR: 15.4) compared to males. In males, higher odds of having WB were found in chickens with a high daily weight gain (OR: 1.75) compared to those with an intermediate daily weight gain. In females, higher odds of having SM were associated to a high slaughter weight (OR: 2.10) while lower odds to a low slaughter weight (OR: 0.87). As for meat quality, only WB meat was clearly different for some technological and sensorial properties, which can play a major role also in meat processing. In conclusion, our analysis identified breast development as a potential risk factor for WS, while a high growth was the risk factor for WB and SM. A different probability of having WB or SM in females and male was found.

## 1 Introduction

Among the most common animal food consumed worldwide due to its nutritional profile, as part of a well equilibrated (vegetable-rich) diet, poultry meat is linked to a lower risk of developing non-communicable diseases, including obesity, cardiovascular diseases, and type 2 diabetes mellitus (Marangoni et al., 2015). This issue supports the development of poultry production, one of the fastest-growing agricultural subsectors, especially in developing areas where it contributes to food security and plays a major role in reducing global hunger (Mottet and Tempio, 2017) with an estimated production increase of 16% by 2029.

The expansion of the poultry production has been sustained by the extraordinary growth potential of chickens pushed in genetic selection programs. However, the increasing growth rate during a short life period (5–8 weeks) and the large body size of modern crossbred birds have resulted in cardiovascular diseases, different metabolic and musculoskeletal disorders, including the most recent emerging breast muscle abnormalities, such as white striping (WS), wooden breast (WB) and spaghetti meat (SM) (Baldi et al., 2021; Hartcher and Lum, 2019; Soglia et al., 2021; Che et al., 2022a, b). The WS breasts are characterized by the presence of visible white striations on the surface in the direction of muscle fiber, while a degenerative myopathy at the fiber level is present (Kuttappan et al., 2013; Radaelli et al., 2017). Sometimes, WS is also accompanied by the most recently discovered SM, though this latter can be found independently, as well (Che et al., 2022a). The WB muscles are characterized by pale and bulging hard areas and, at the morphological level, by polyphasic myodegeneration with regeneration and accumulation of interstitial connective tissue or fibrosis (Sihvo et al., 2014; Velleman and Clark, 2015). Finally, the few available data show that SM affects mainly the connective tissue within the perymisial compartments causing the formation of large intracellular spaces and consequently overall impaired integrity of the *Pectoralis major* (Soglia et al., 2021). In SM meat, muscle fibers can be easily separated resembling the long, thin, solid, and cylindrical appearance of spaghetti (Baldi et al., 2018).

Omics analyses have been used to define pathophysiological mechanisms of broiler myopathies with the main focus on WS and WB (Mutryn et al., 2015; Zambonelli et al., 2016; Kuttappan et al., 2017a; Boerboom et al., 2018; Soglia et al., 2020). These studies have suggested that a reduced vascularity and ischemia, with consequent hypoxia and lack of blood flow, lead to WB. Results of RNA-seq analysis support localized hypoxia, oxidative stress, increased intracellular calcium, and the possible presence of muscle fiber-type switching, as key features of WB (Mutryn et al., 2015). Furthermore, Zambonelli et al. (2016) found that breasts with WS and WB have an increased expression of genes associated with metabolic oxidative stress, inflammation, regeneration, glucose metabolism, lipidosis, fibrosis, and proteoglycan synthesis. Recently, Bordini et al. (2021) suggested that alterations in extracellular matrix composition could somehow activate the cascade of biological reactions that result in the growth-related myopathies onset, and hypothesized the involvement of Collagen IV alterations in activating the endoplasmic reticulum stress response.

From published data, it is not completely clear what factors influence the manifestation of a particular myopathy and neither what the initial trigger is (Bailey et al., 2020). Previous studies highlighted the importance of understanding the environmental and/or management factors that contribute greater than 65% of the variance in the incidence of WS and more than 90% of the variance of the incidence of WB (Bailey et al., 2015). This was stressed in recent analyses of risk factors for myopathies that collected data from different flocks in two Canadian slaughterhouses (Che et al., 2022b) which identified high environmental temperature during the grow-out period and absence of vaccination against coccidian as risk factors for increased probability of showing SM and WB, respectively.

Some studies suggest that there are biological characteristics in male broilers that make them more susceptible to WB (Brothers et al., 2019), while female broilers have a higher predisposition for SM (Pascual et al., 2020b). Other studies suggest that growth and carcass traits, in particular breast weight, are predominant factors for WS and WB occurrence (Aguirre et al., 2020; Santos et al., 2021). In addition, there is published information for the effects of feeding strategies, such as feed restriction (Trocino et al., 2015; Radaelli et al., 2017; Gratta et al., 2019) and time-limited feeding (Livingston et al., 2019), or the supplementation of sodium butyrate (Pascual et al., 2020b), guanidinoacetic acid (Córdova-Noboa et al., 2018a; Córdova-Noboa et al., 2018b), selenium (Cemin et al., 2018) and microalgae (Khan et al., 2021) on myopathies occurrence. Research has also focused on the effect of decreasing amino acid density to control growth and reduce the rates and degrees of myopathies (Cruz et al., 2017; Bodle et al., 2018; Meloche et al., 2018).

While quality of defective meat has been extensively compared within several studies, results among studies are often not consistent about changes in traits associated to *post mortem* metabolism and in rheological or sensorial properties of meat (i.e., pH, water retention, colour and hardness). Available data have been produced both using samples collected in controlled experimental trials and in commercial slaughterhouses from commercial flocks. Few studies investigated the correlation between individual growth traits and myopathy occurrence (Bailey et al., 2015; Aguirre et al., 2020; Santos et al., 2021). Also, according to our knowledge, only one study included SM in such analyses using flock data (Che et al., 2022b).

Thus, the present study aimed at elucidating the role of some major factors individually related to chickens (sex, growth and carcass traits) on the occurrence of myopathies, besides at characterizing rheological traits of defective meat, using a set of data collected from 2015 to 2021 within trials conducted in the controlled facilities of the University of Padova (Department of Agronomy, Food, Natural resources, Animals and Environment) using the same reference and lab methods under standardized conditions.

## 2 Materials and Methods

### 2.1 Dataset

The present study used the data collected on 1,278 carcasses and 890 breasts obtained from eight trials performed at the University of Padova that used a total of 6,036 broiler chickens (Table 1). At commercial slaughtering the carcasses were used for scoring myopathy occurrence and the breasts were submitted to rheological analyses for meat quality (Supplementary Tables S1, S2).

**TABLE 1:** Summary of the main information about the eight experimental trials originating the dataset.

Trial	References	Main Experimental Factors	Sex	Genotype	Slaughtering Age, d	Recordings, n		
						Growth performance	Breast weight and myopathy occurrence	Meat quality traits
1	Trocino et al. (2015)	Early feed restriction from 13 to 21 days	Females and males	A, B	46	768	127	128
2	Gratta et al. (2019)	Early vs late feed restriction	Males	B, C	48	900	216	108
3	Not published yet	Light programs	Females and males	B, C	44	800	191	95
4	Pascual et al. (2020b)	Dietary supplementation with sodium butyrate	Females and males	B	45	768	192	96
5	Pascual et al. (2020a)	Dietary supplementation with yeast cell wall extracts	Males	B	44	576	120	72
6	Not published yet	Dietary supplementation with grape pomace extracts	Females	B	41	560	160	120
7	Pascual et al. (2022)	Dietary supplementation with grape pomace and chestnut extracts	Females and males	B	45	864	144	144
8	Huerta et al. (2022)	Dietary supplementation with grapeseed extracts	Females and males	B	42	800	128	128

Genotype A, Ross 708; Genotype B, Ross 308; Genotype C, Cobb 500.

The trials used three commercial genotypes selected for fast growth and high-breast yield: genotype B (Ross 308) was the mostly used (eight trials), genotype A (Ross 708) was used only in one trial, and genotype C (Cobb 500) in two trials (Table 1). Five trials used chickens of both sexes, half females and half males within trial; two trials used only males and one trial only females. Slaughtering age ranged from 41 to 42 days in two trials to 44–45 days in four trials, and 46–48 days in the remaining two trials. A variety of experimental factors was tested in the different trials which were directly addressed to control the occurrence of myopathies (e.g., feeding or management strategies aimed at reducing growth rate based on feed restriction; light management) or aimed at the use of different feed additives to improve gut health of broiler chickens with potential implication on myopathies occurrence (e.g., butyrate, yeast cell wall extracts, grape by-product extracts) (Table 1).

### 2.2 Growth Data Recordings

All trials were conducted at the Experimental Farm of the University of Padova (Legnaro, Padova, Italy) in a poultry house equipped with cooling system, forced ventilation, radiant heating, and controlled light systems. Broiler chickens were kept in wire-net pens with a concrete floor covered with wood shaving litter, each equipped with nipple drinkers and circular feeders for manual distribution of feed. Usually, a total of 24 h of light was provided during the first 2 days after the arrival of the chicks. Subsequently, the hours of light were progressively reduced until an 18L:6D photoperiod was achieved, which was then maintained from 12 to 13 days of age onward. Chicks were individually identified by a plastic band at the leg; they were always weighed on the day of their arrival and at the end of the trial for obtaining daily weight gain. Feed consumption was controlled on a pen level for which nor feed intake or feed conversion rate were used for the purposes of the present study.

### 2.3 Commercial Slaughtering and Carcass and Meat Quality Recordings

At the end of the trials, all chickens were slaughtered in a single commercial slaughterhouse, which was close to the experimental farm, therefore guaranteeing homogeneous slaughtering procedures and carcass preparation. Ready-to-cook carcasses were recovered after 2 h of refrigeration at 2°C and individually weighed to measure the slaughter dressing percentage. In all trials, a number of carcasses, previously selected on the basis of the final live weight as corresponding to the mean body weight within a pen, were subjected to gross examination between 8 and 24 h after slaughtering to evaluate the occurrence (presence or absence) in *pectoralis major* muscles of i) white striping (either moderate or severe) (Kuttappan et al., 2012a); ii) wooden breast (firm upon palpation, prominent ridge like bulge on caudal area of fillet, clear viscous fluid cover and/or petechial multifocal lesions on the fillet surface) (Sihvo et al., 2014); iii) spaghetti meat exhibiting an overall impaired integrity and tendency toward separation of the muscle fiber bundles especially within the cranial part of the fillet) (Baldi et al., 2018). Carcasses were stored at 2°C for 24 h after slaughtering and then dissected in the main cuts (breast, wings, thighs, and drumsticks). *Pectoralis major* muscles were separated from the breasts: the pH of the *p. major* muscles were measured in triplicates on their ventral side with a pH meter (Basic 20, Crison Instruments Sa, Carpi, Italy) equipped with a specific electrode (cat. 5232, Crison Instruments Sa, Carpi, Italy); the L*a*b* color indexes were measured in triplicate on the ventral side of the same muscles using a Minolta CM-508 C spectrophotometer (Minolta Corp., Ramsey, NJ, United States). After then, one meat portion (8 cm × 4 cm × 3 cm) was separated from the cranial side of the *p. major* muscle, parallel to the direction of the muscle fibers, and processed for the following analyses after storage under vacuum in plastic bags at −18 °C. Thawing and cooking losses were measured in this cut (Petracci and Baéza 2011). Meat cuts (fresh or thawed depending on the trials) were individually placed in plastic bags and cooked in a water bath until an internal temperature of 80°C was achieved. After 40 min of cooling, another meat portion (4 cm × 2 cm × 1 cm) was separated from the cooked one to assess the maximum shear force using an LS5 dynamometer (Lloyd Instruments Ltd., Bognor Regis, United Kingdom) using the Allo-Kramer (10 blades) probe (load cell: 500 kg; distance between the blades: 5 mm; thickness: 2 mm; cutting speed: 250 mm/min) (Mudalal et al., 2015).

### 2.4 Statistical Analyses

Data were analyzed using SAS 9.4 (Statistical Analysis System, 2013). Descriptive statistics of growth and slaughter traits, myopathy occurrence and breast quality traits were obtained using PROC FREQ and PROC MEANS procedures.

To identify the risk factors related to myopathy occurrence, the effects of sex, genotype, growth traits (daily weight gain and slaughter weight) and slaughter traits (breast weight and breast yield) were evaluated by univariate and multivariate logistic regression analysis using the PROC LOGISTIC of SAS. Initially, variables were screened for multicollinearity (correlation coefficient |r|<0.7). A univariate analysis was performed for each independent variable and for each myopathy. Given the strong influence of sex on growth and slaughter traits of chickens, we firstly evaluated sex as a potential influencing factor on breast myopathy occurrence (Supplementary Table S3). Then, data were assigned to three classes of growth traits and slaughter traits with a similar number of data (33% of the available data set) within each sex, distinguishing between low, intermediate, and high growth traits or slaughter traits. Finally, we evaluated the effects of genotype and classes for daily growth traits or slaughter traits within each sex (Supplementary Tables S4–S6). Thereafter, independent variables that showed a *p* < 0.05 in the univariate analysis were included into a multivariate logistic regression analysis and the risk factors were identified through a forward stepwise selection based on *p* < 0.05. The regression coefficients were expressed as odds ratio (OR) with 95% confidence interval (CI). The evaluation of risk factors within each genotype was also performed and results are shown in Supplementary Tables S7–S9.

Individual data of pH, colour, cooking losses and shear force measured on *p. major* were submitted to ANOVA with myopathy, sex, and their interactions as the main effects and the trial as a random effect, using the PROC MIXED. Differences among means with *p* < 0.05 were assumed to be statistically significant. Differences among means with 0.05 < *p* < 0.10 were regarded as approaching significance.

## 3 Results

### 3.1 Descriptive Statistics of Growth, Carcass Data and Meat Quality Traits

Descriptive statistics for males and females and for the three genotypes are provided in Tables 2 and 3, respectively.

**TABLE 2:** Descriptive statistics of growth traits, slaughter traits, myopathy occurrence and meat quality of *pectoralis major* in broiler chickens: data collected over eight experimental trials.

	All Chickens						Females						Males					
	No	Av	SD	Min	Max	CV	No	Av	SD	Min	Max	CV	No	Av	SD	Min	Max	CV
Growth and slaughter traits																		
Daily weight gain, g/d	1,278	70.7	7.94	53.3	92.5	11.2	549	66.1	6.01	53.3	85.9	9.09	729	74.3	7.43	57.4	92.5	10.0
Slaughter weight, g	1,278	3,154	401	2,186	4,260	12.7	549	2,873	309	2,186	3,887	10.8	729	3,368	323	2,561	4,260	9.59
Breast weight, g	930	912	122	560	1,258	13.4	453	835	83.1	560	1,112	9.95	477	986	107	721	1,258	10.9
Breast yield, %	930	40.0	2.05	32.9	46.4	5.13	453	40.7	1.88	32.9	45.1	4.62	477	39.3	1.97	32.9	46.4	5.01
Myopathies occurrence^a^																		
White Striping, %	8	64.7	11.6	44.5	77.0	17.9	6	60.0	12.5	37.1	70.5	20.8	7	68.6	12.2	52.3	83.3	17.8
Wooden breast, %	8	18.6	10.6	5.09	36.1	57.0	6	12.1	5.1	3.16	17.5	42.2	7	25.2	17.6	5.09	55.6	69.8
Spaghetti meat, %	8	13.0	14.1	5.00	41.9	108	6	36.7	10.6	25.0	48.6	28.9	7	3.48	1.50	1.56	5.04	43.1
Breast quality traits																		
pH	890	5.92	0.15	5.37	7.04	2.53	413	5.93	0.12	5.57	6.32	2.02	477	5.91	0.16	5.37	7.04	2.71
L*	890	47.2	3.11	38.3	55.5	6.59	413	47.5	2.78	41.2	55.5	5.85	477	47.0	3.37	38.3	54.8	7.17
a*	890	1.25	1.75	-2.50	5.85	140	413	1.34	1.63	-1.98	4.63	122	477	1.16	1.77	-2.50	5.85	153
b*	890	17.2	5.11	5.48	32.4	29.7	413	17.5	4.82	6.36	27.4	27.5	477	17.0	5.34	5.48	32.4	31.4
Thawing losses, %	818	9.75	3.07	1.04	26.7	31.5	339	10.1	3.00	3.33	19.9	29.7	334	9.44	3.12	1.04	26.7	33.1
Cooking losses, %	818	26.2	5.67	15.6	45.2	21.6	378	26.9	6.78	15.6	45.2	25.2	440	25.5	4.42	15.9	41.1	17.3
Shear force, kg/g	818	3.10	1.02	1.44	12.2	32.9	377	3.01	0.77	1.76	7.32	25.6	440	3.18	1.20	1.44	12.2	37.7

No, number of chickens; Av, Average; SD, standard deviation; Min, Minimum; Max, Maximum; CV, coefficient of variation.

a Occurrence of myopathies within the trials. Not exclusive myopathy, i.e. white striping, wooden breast and/or spaghetti meat can be associated in the same breast.

**TABLE 3:** Descriptive statistics of growth traits, slaughter traits, myopathy occurrence and meat quality of *pectoralis major* in broiler chickens from three different genotypes: data collected over eight experimental trials.

	Genotype A						Genotype B						Genotype C					
	No	Av	SD	Min	Max	CV	No	Av	SD	Min	Max	CV	No	Av	SD	Min	Max	CV
Growth and slaughter traits																		
Daily weight gain, g/d	63	69.7	7.40	56.8	83.8	10.6	1,011	70.5	8.10	53.3	92.0	11.5	204	72.2	7.13	55.1	92.5	9.88
Slaughter weight, g	63	3,205	340	2,613	3,854	10.6	1,011	3,117	409	2,186	4,260	13.1	204	3,327	322	2,426	4,070	9.67
Breast weight, g	63	920	134	609	1,191	14.6	765	900	118	560	1,258	13.2	102	998	108	743	1,224	10.8
Breast yield, %	63	39.9	2.12	32.9	43.9	5.32	765	39.9	2.01	32.9	45.1	5.05	102	40.7	2.16	36.1	46.4	5.31
Myopathies occurrence^a^																		
White Striping, %	1	49.2	-	-	-	-	8	64.6	13.1	40.6	78.7	20.3	2	74.1	2.7	72.2	76.0	3.6
Wooden breast, %	1	14.2	-	-	-	-	8	18.7	10.4	6.32	36.1	55.7	2	5.00	1.80	3.70	6.25	36.2
Spaghetti meat, %	1	-	-	-	-	-	8	20.7	14.1	5.00	41.9	67.7	2	-	-	-	-	-
Breast quality traits							8											
pH	63	5.83	0.10	5.62	6.04	1.69	725	5.94	0.14	5.49	7.04	2.34	102	5.84	0.16	5.37	6.24	2.78
L*	63	46.1	2.27	41.3	52.3	4.91	725	47.6	3.08	39.1	55.5	6.46	102	44.8	2.56	38.3	51.1	5.71
a*	63	−0.97	0.59	−2.50	0.28	60.7	725	1.58	1.69	−1.6	5.85	107	102	0.27	0.66	−1.40	1.89	248
b*	63	13.1	1.97	9.01	19.0	15.0	725	17.8	5.40	5.48	32.4	30.3	102	16.0	2.14	10.4	20.7	13.4
Thawing losses, %	63	8.47	3.28	3.10	26.7	38.7	507	10.0	3.09	1.04	19.9	30.8	102	9.14	2.57	4.21	15.5	28.1
Cooking losses, %	63	23.8	3.28	18.6	35.3	13.8	653	26.4	6.08	15.6	45.2	23.0	102	25.8	3.30	21.2	36.3	12.8
Shear force, kg/g	63	2.99	1.05	1.92	6.69	35.2	653	3.19	1.06	1.44	12.2	33.3	102	2.60	0.48	1.64	4.54	18.4

No: number of chickens; Av: Average; SD: standard deviation; Min: Minimum; Max: Maximum; CV: coefficient of variation genotype A: Ross 708; genotype B: Ross 308; genotype C: Cobb 500.

a Occurrence of myopathies within the trials. Not exclusive myopathy, i.e. white striping, wooden breast and/or spaghetti meat can be associated in the same breast.

In the whole data set, individual daily weight gain of broiler chickens ranged from 53.5 g/d to 92.5 g/d, averaging at 70.7 g/d for a coefficient of variation (CV) at 11.2%. This growth corresponded to an average slaughter weight of 3,154 g (CV: 12.7%), a breast weight (with bone) of 912 g (CV: 13.4%) and a breast yield as a proportion of the carcass of 40.0% with a low variability (CV: 5%) (Table 2). While average daily weight gain, slaughter weight and breast weight were lower in females than in males, breast yield between the two sexes was rather similar (on average, 40.7% in females and 39.3% in males).

Breasts exhibiting WS (alone or in associations with other myopathies) averaged at 64.7% with minimum and maximum occurrence at 44.5% and 77.0%, respectively (Table 2). The occurrence of both WB and SM averaged at 11%–12% with a large variability (CV: 57.0% for WB and 108% for SM) which depended on differences between females and males. In fact, WB occurred more in males (average of trials 25.2% with a range from 5.09% to 55.6%) than in females (average of trials 12.1%, range 3.16%–17.5%), while SM showed an opposite trend (females: average of trials 36.7%, range 25.0%–48.6%; males: average 3.48%, range 1.56%–5.04%).

As for meat quality, the less variable traits in the whole data set and in the sub-sets per sex were final pH (CV: 2%–3%) and lightness (CV: 6%–7%), while meat water holding capacity (measured by means of thawing and cooking losses), texture (measured as shear force), and the colour indexes of redness (a*) and yellowness (b*) showed a large variability (Table 2). On the whole, no main differences in average values or in variability for meat quality traits were recorded between males and females.

### 3.2 Risk Factors for Breast Myopathy Occurrence

Potential risk factors influencing breast myopathy occurrence in broiler chickens were firstly identified based on a univariate logistic regression analysis and then included into the multivariate regression model. Based on the univariate analysis, potential influencing factors for WS occurrence were sex, genotype, daily weight gain, slaughter weight, breast weight, and breast yield (Supplementary Tables S3, S4). As for WB, potential influencing factors were sex, genotype, and daily weight gain (Supplementary Table S5), while sex, daily weight gain, and slaughter weight were identified as risk factors for SM (Supplementary Table S6).

Results from the multivariate logistic regression analysis of the factors selected for WS occurrence are reported in Table 4. Genotype, breast weight, and breast yield significantly influenced WS occurrence. Regarding the first factor, genotype C had higher odds of WS occurrence than genotype A (OR: 2.75; 95% CI: 1.37–5.52). As for breast traits, compared to intermediate-weight breasts (850–960 g), chickens with a high-weight breast (>960 g) showed higher odds of WS occurrence (OR: 1.49; 95% CI: 1.03–2.15), whereas those with a low-weight breast (<850 g) showed lower odds (OR: 0.57; 95% CI: 0.41–0.80). A similar trend was found for breast yield: compared to intermediate-yield breasts (39.0–40.7%), chickens with high-yield breasts showed higher odds of WS occurrence (OR: 1.27, 95% CI: 0.89–1.81) whereas those with low-yield breasts showed lower odds (OR: 0.82; CI: 0.58–1.15) (Table 4).

**TABLE 4:** Factors influencing white striping occurrence in broiler chickens extracted by forward selection in the multivariate logistic regression analysis.

Variable	Estimate	SE	Odds Ratio	95% CI		*p value*
				Lower	Upper	
Intercept	0.55	0.12	-	-	-	<0.001
Genotype						
A (Ref)	-	-	-	-	-	-
B	0.18	0.13	2.18	1.28	3.71	0.16
C	0.41	0.19	2.75	1.37	5.52	<0.05
Breast weight						
Intermediate: 850–960 g (Ref)	-	-	-	-	-	-
Low: <850 g	−0.50	0.10	0.57	0.41	0.80	<0.001
High: >960 g	0.45	0.11	1.49	1.03	2.15	<0.001
Breast yield						
Intermediate: 39.0–40.7% (Ref)	-	-	-	-	-	-
Low: <39.0% g	−0.21	0.10	0.82	0.58	1.15	<0.05
High: >40.7%	0.22	0.10	1.27	0.89	1.81	<0.05

SE, standard error; CI, confidence interval; Ref, reference. Genotype A, Ross 708; Genotype B, Ross 308; Genotype C, Cobb 500.

**FIGURE 1:**
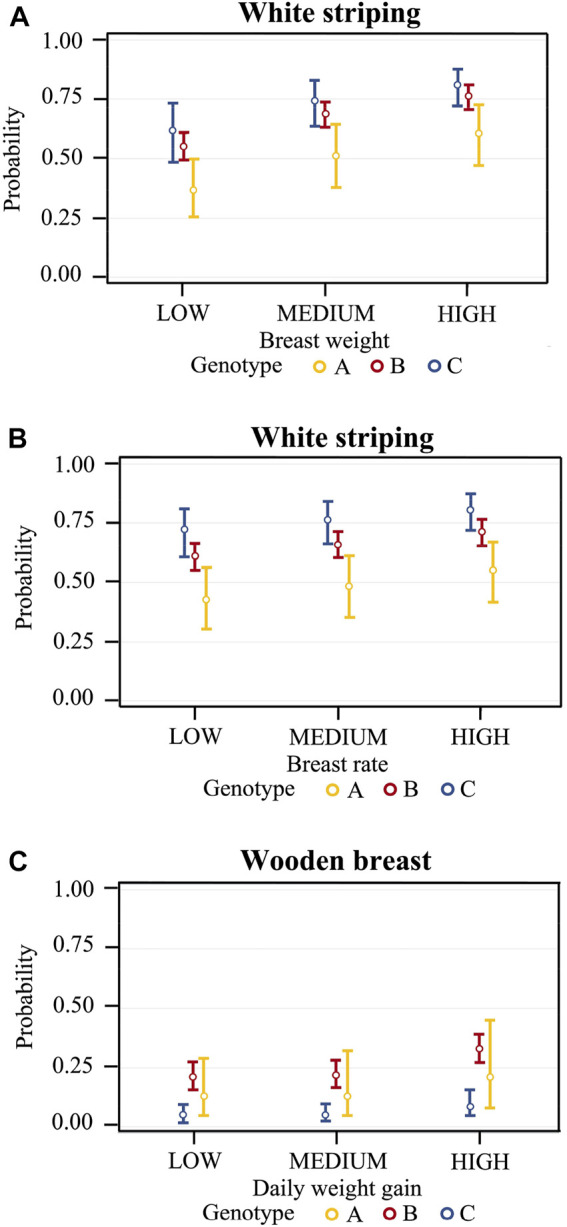
Predicted probabilities of WS occurrence for breast weight class (LOW <850 g, MEDIUM 850–960 g, HIGH >960 g) in male and female broiler chickens **(A)**; predicted probabilities of WS occurrence for breast yield class LOW <39.0%, MEDIUM 39.0–40.7%, HIGH >40.7%) **(B)**; predicted probabilities of WB occurrence for daily weight gain class LOW <70.7 g/d, MEDIUM 70.7–78.0 g/d, HIGH >78.0 g/d) **(C)**. Predicted probabilities are given separately for the different genotypes (genotype A: Ross 708; genotype B: Ross 308; genotype C: Cobb 500) used in the experimental trials at the University of Padova.

The representation of the significant effects of genotype within breast weight classes showed that the predicted probabilities of WS occurrence in chickens with low-weight breasts were 42.5% (95% CI: 30.1%–55.9%), 60.5% (95% CI: 54.8%–66.1%), and 72.0% (95% CI: 60.6%–81.1%) for genotype A, B, and C respectively (Figure 1A). Then, in chickens with intermediate-weight breasts, the probabilities were 48.3% (95% CI: 35.4%–61.4%), 66.0% (95% CI: 60.3%–71.3%), and 76.5% (95% CI: 66.1%–84.4%) for the same genotypes. Finally, in chickens with high-weight breasts, the probabilities were 54.8% (95% CI: 41.7%–67.3%), 71.6% (95% CI: 66.1%–76.5%), and 80.8% (95% CI: 72.0%–87.4%). As for breast yield (Figure 1B), the predicted probabilities of WS occurrence in chickens with low yields were 36.7% (95% CI: 25.3%–49.8%), 55.0% (95% CI: 49.3%–60.5%), and 61.6% (95% CI: 48.4%–73.3%) for genotype A, B and C, respectively; in chickens with intermediate yields the probabilities were 51.4% (95% CI: 38.0%–64.6%), 69.0% (95% CI: 63.6%–74.0%), and 74.6% (95% CI: 63.4%–83.2%), while in chickens with high yields the probabilities were 60.5% (95% CI: 47.0%–72.6%), 76.4% (95% CI: 70.8%–81.2%), and 81.0% (95% CI: 72.3%–87.4%).

Regarding the risk factors for WB occurrence, since the results of the univariate analysis showed that females had lower odds of having WB (OR: 0.55; 95% CI: 0.40–0.75) (Supplementary Table S3), the multivariate logistic regression analysis used only data from males (Table 5). In these latter chickens, WB occurrence was significantly influenced by genotype and daily weight gain. Compared to genotype A, genotype B showed higher odds (OR: 1.85; 95% CI: 0.61–5.58) and genotype C lower odds of having WB (OR: 0.35; 95% CI: 0.10–1.26). Males with a high daily weight gain (>78 g/d) showed higher odds of having WB (OR: 1.75; 95% CI: 1.12–2.73) compared with males with an intermediate daily weight gain (70.7–78.0 g/d), whereas no significant differences were found between males with intermediate and low daily weight gain (Table 5).

**TABLE 5:** Factors influencing wooden breast occurrence in male broiler chickens and extracted by forward selection in the multivariate logistic regression analysis.

Variable	Estimate	SE	Odds Ratio	95% CI		*p value*
				Lower	Upper	
Intercept	−1.87	0.22	-	-	-	<0.001
Genotype						
A (Ref)	-	-	-	-	-	-
B	0.76	0.23	1.85	0.61	5.58	<0.001
C	−0.90	0.30	0.35	0.10	1.26	<0.01
Daily weight gain						
Intermediate: 70.7–78.0 g/d (Ref)	-	-	-	-	-	-
Low: <70.7 g/d	−0.22	0.15	0.95	0.58	1.56	0.12
High: >78.0 g/d	0.39	0.13	1.75	1.12	2.73	<0.01

SE, standard error; CI, confidence interval; Ref, reference. Genotype A, Ross 708; Genotype B, Ross 308; Genotype C, Cobb 500.

The representation per genotype within classes of daily weight gain (low, intermediate, and high) showed that the predicted probabilities of WB occurrence in male chickens with low daily weight gain were 12.5% (95% CI: 4.76%–28.9%), 20.8% (95% CI: 15.4%–27.5%) and 4.76% (95% CI: 2.32%–9.51%) for genotype A, B, and C respectively (Figure 1C). In males with intermediate daily weight gain, the probabilities were 12.5% (95% CI: 4.87%–28.9%), 21.7% (95% CI: 16.4%–28.2%), and 5.01% (95% CI: 2.50%–9.77%) for the same genotypes A, B, and C, while in chickens with a high daily weight gain the probabilities were 18.7% (95% CI: 8.68%–43.8%), 32.7% (95% CI: 26.7%–39.4%), and 8.46% (95% CI: 4.31%–15.93%), respectively.

Regarding the risk factors for SM occurrence, since the results of the univariate analysis showed that females had significantly higher odds of having SM (OR: 15.4; 95% CI: 8.55–27.8) (Supplementary Table S3), the multivariate logistic regression analysis used only data from females (Table 6). In these latter, SM occurrence was significantly influenced only by slaughter weight. In fact, compared with females with an intermediate slaughter weight (2,720 g to 2,935 g), chickens with a high slaughter weight (>2,935 g) showed higher odds of having SM (OR: 2.10; 95% CI: 1.18–3.80), whereas those with a low slaughter weight (<2,720 g) showed lower odds (OR: 0.87; 95% CI: 0.53–1.42).

**TABLE 6:** Factors influencing spaghetti meat occurrence in female broiler chickens and extracted by forward selection in the multivariate logistic regression analysis.

Variable	Estimate	SE	Odds Ratio	95% CI		*p* value
				Lower 95%	Upper 95%	
Intercept	−0.30	0.12	-	-	-	<0.05
Slaughter weight						
Intermediate: 2,720–2,935 g (Ref)	-	-	-	-	-	-
Low: <2,720 g	−0.34	0.16	0.87	0.53	1.42	<0.05
High: >2,935 g	0.54	0.18	2.10	1.18	3.80	<0.01

SE, standard error; CI, confidence interval; Ref, reference.

The results of the risk factor analysis within each genotype are reported in Supplementary Tables S7–S9. In male and female broiler chickens, WS occurrence was significantly influenced by breast weight in genotype B, and by breast weight and breast rate in genotype C (Supplementary Table S7). In males, WB was affected by breast rate in genotype B (Supplementary Table S8). In females, SM occurrence was influenced by slaughter weight both in genotype A and B (Supplementary Table S9).

### 3.3 Meat Quality

We firstly evaluated the effect of the presence of any myopathy (SM, WB, and WS alone or in combination) and the effect of sex on meat rheological traits (Table 7). Final pH, shear force, and thawing and cooking losses measured on *p. major* were significantly different in normal meat compared to defective meat, while colour (i.e. lightness, yellowness and redness indices) was not affected. In details, compared to normal meat, the pH of defective meat was significantly higher (*p* < 0.01), as it was for shear force (*p* < 0.001) and cooking losses (*p* < 0.001). Moreover, most traits were significantly different in females compared to males, except for the redness index and thawing losses. In details, breasts of males displayed higher pH, cooking losses, and lightness, while the yellowness index was higher in females (Table 7). No significant interaction between the presence of myopathy and the sex of chickens was measured.

**TABLE 7:** Effect of myopathy presence, sex and their interactions on breast quality traits.

	Myopathy (M)		Sex (S)		*p* value			RMSE
	Absent	Present	Male	Female	M	S	M×S	
Chickens, n	354	924	729	549	-	-	-	-
pH	5.90	5.92	5.93	5.89	<0.01	<0.001	0.50	0.12
Lightness (L*)	47.2	47.3	47.4	47.0	0.36	<0.05	0.15	2.33
Redness (a*)	1.38	1.45	1.32	1.45	0.15	0.45	0.94	0.64
Yellowness (b*)	17.9	18.0	17.8	18.1	0.94	<0.05	0.53	1.80
Thawing losses, %	9.32	9.70	9.49	9.70	0.11	0.87	0.90	2.58
Cooking losses, %	25.0	26.6	26.8	24.8	<0.001	<0.001	0.12	4.17
Total losses, %	33.9	36.1	35.9	34.1	<0.001	<0.001	0.56	4.77
Shear force, kg/g	2.92	3.26	3.18	3.00	<0.001	<0.05	0.94	0.90

**FIGURE 2:**
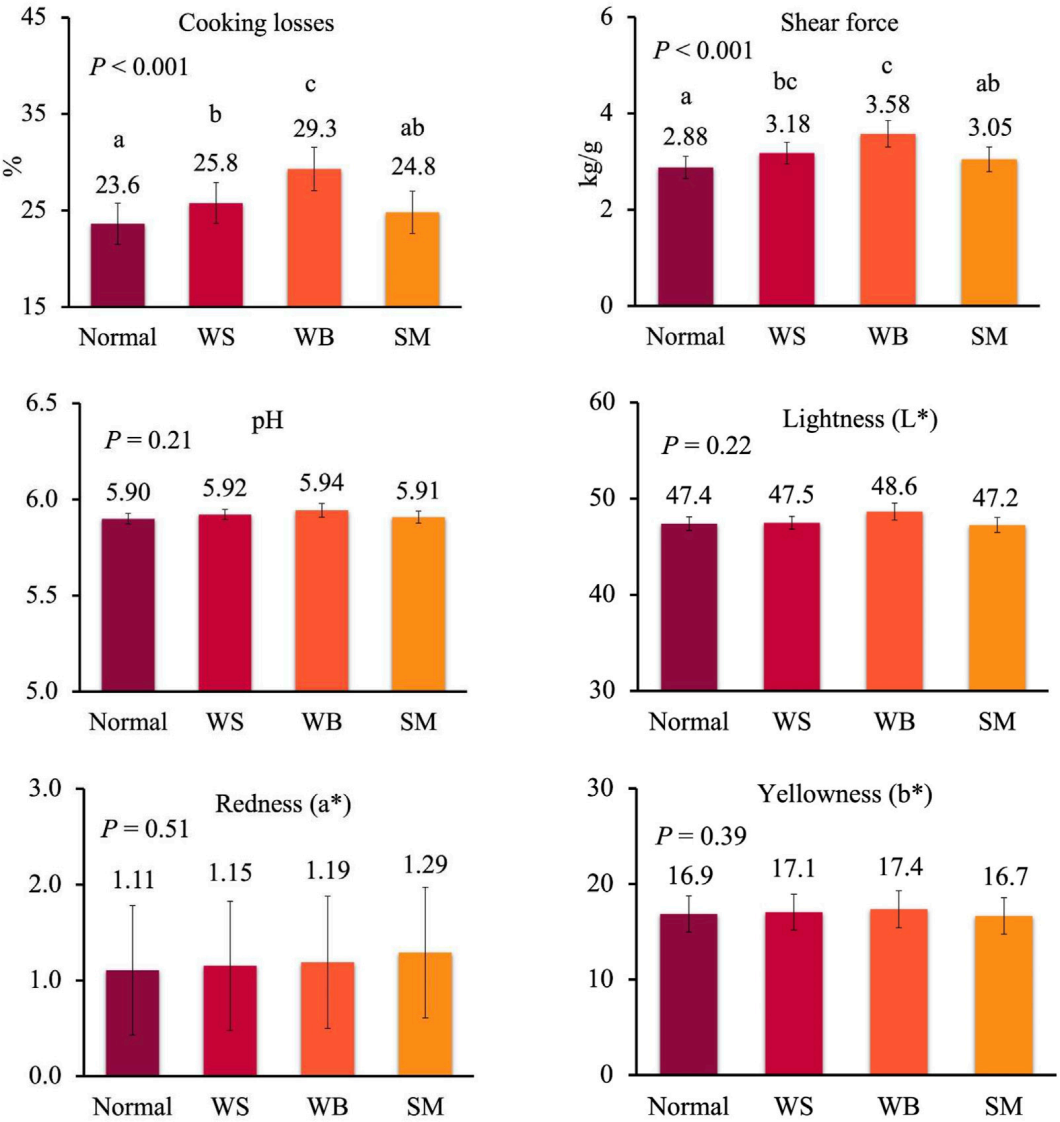
Meat quality in normal breasts and in breasts affected by wooden breast (WB), white striping (WS) and spaghetti meat (SM) of female broiler chickens (samples with only one myopathy). Data are presented as LS means ± standard error. Different letters above bars indicate significant differences between LS means. [Number of chickens per group available in Supplementary Table S2].

**FIGURE 3:**
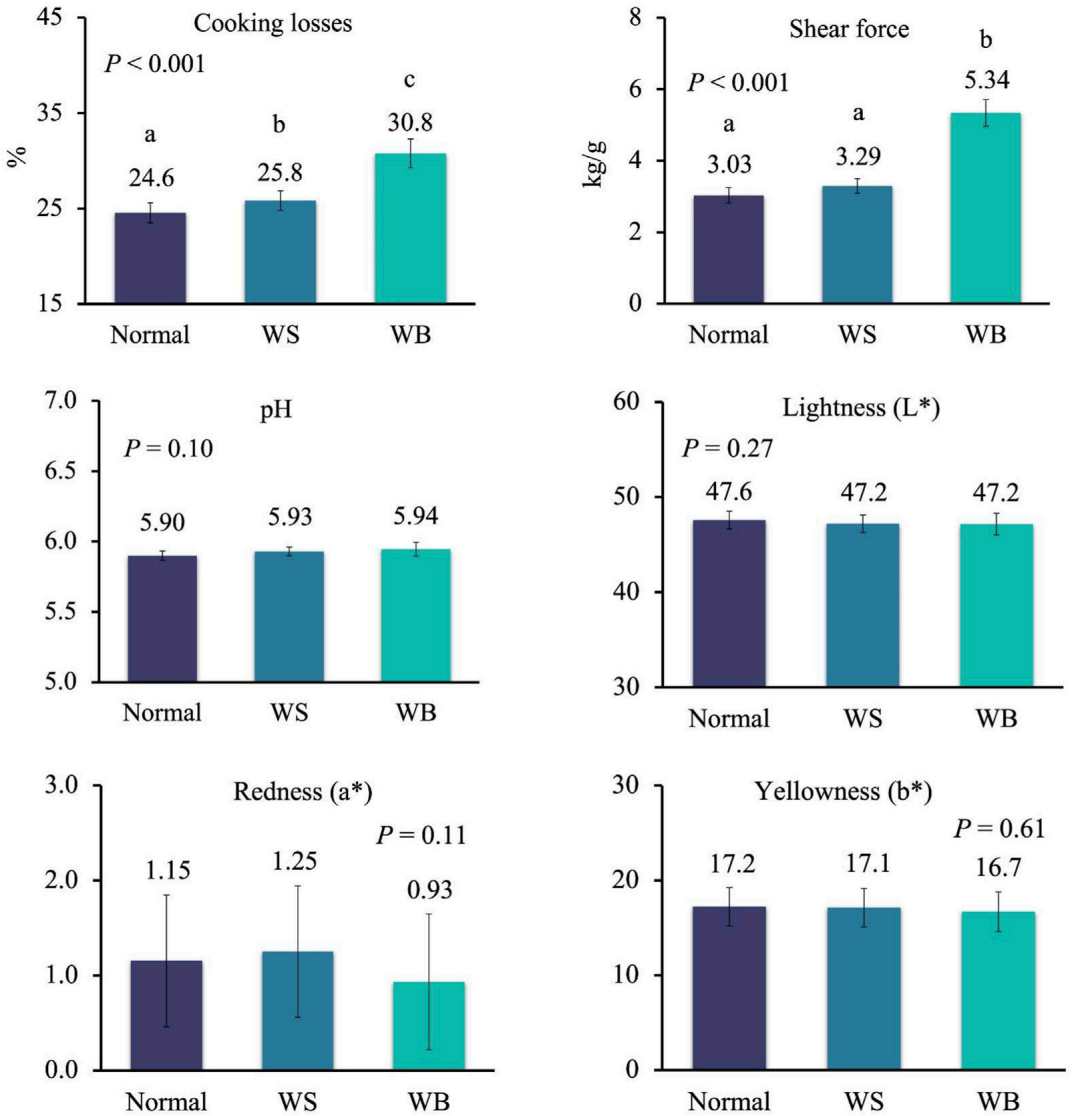
Meat quality in normal breasts and in breasts affected by white striping (WS) and wooden breast (WB) of male broiler chickens (samples with only one myopathy). Data are presented as LS means ± standard error. Different letters above bars indicate significant differences between LS means [Number of chickens per group available in Supplementary Table S2].

To get deeper insight in the effect of the single myopathies on meat quality, data from female and male chickens affected by one myopathy per time were analysed separately because of the large differences in the occurrence of WB and SM in the two sexes, as discussed above. In females, cooking losses significantly increased from normal to WS to WB breasts, while these losses were similar between normal and SM breasts and between WS and SM breasts (Figure 2; *p* < 0.001). A similar trend was observed for shear force which significantly increased from normal to WS and WB breasts (2.88–3.18 and 3.58 kg/g; *p* < 0.001), whereas no difference was recorded between normal and SM breasts and between WS and SM breast (*p* < 0.001) (Figure 2). When data of males affected by a single myopathy were compared (Figure 3), cooking losses significantly increased from normal breasts to WS and, especially, WB ones (24.6% to 25.8% to 30.8%; *p* < 0.001) which corresponded to a higher shear force measured on WB compared to normal and WS breasts (5.34 kg/g vs. 3.03 and 3.29 kg/g; *p* < 0.001). Some trends approaching statistical significance were recorded for pH–which tended to be lower in normal meat compared to defective meat (5.90 vs 5.93–5.94 in normal vs WS and WB meat; *p* = 0.10)—and for redness index–which tended to be higher in normal and WS meat compared to WB meat (1.15 and 1.25 vs 0.93; *p* = 0.11).

**FIGURE 4:**
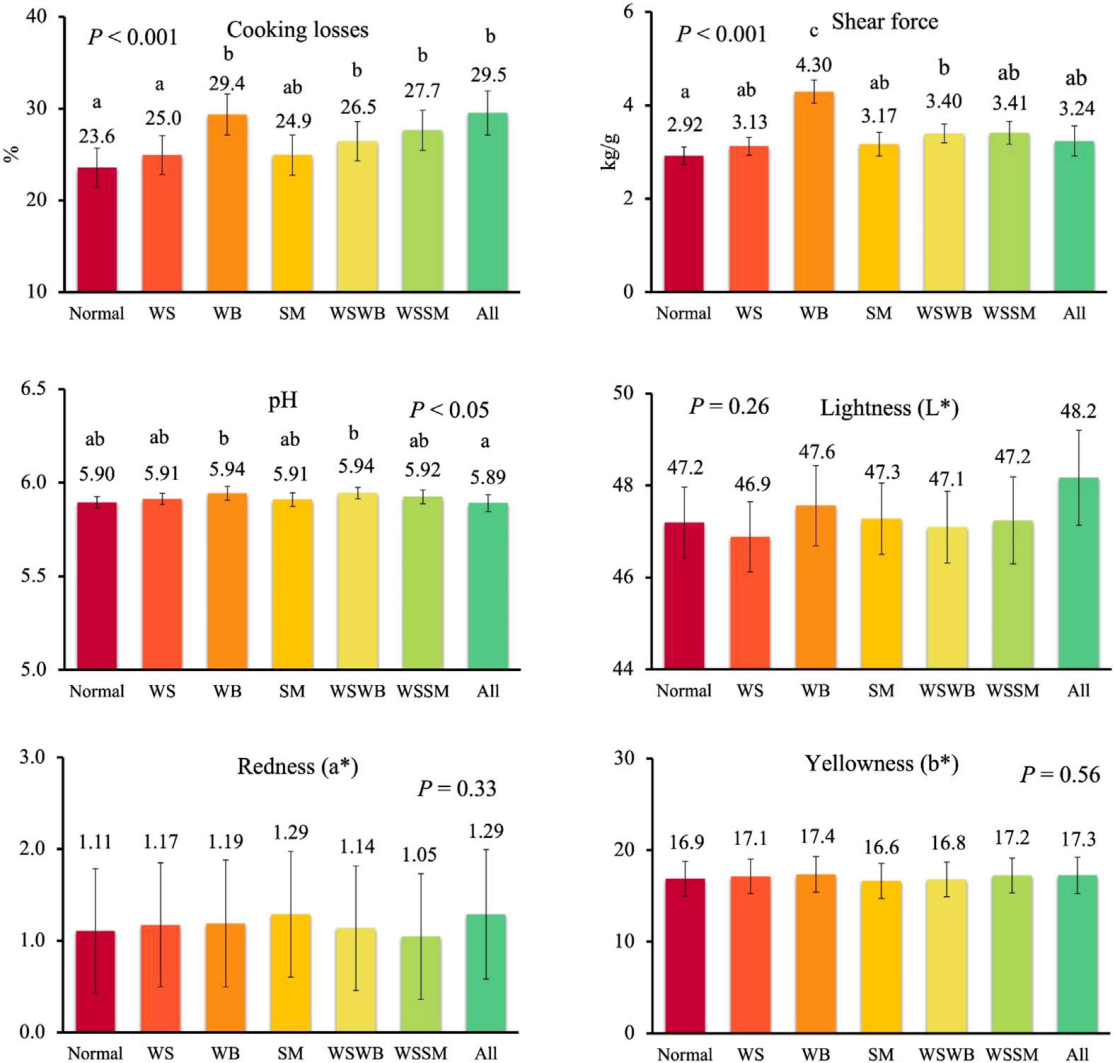
Meat quality in normal breasts and in breasts affected by exclusively white striping (WS) or wooden breast (WB) or spaghetti meat (SM), or contemporarily all myopathies (All), or WS and SM, or WS and WB. Data are presented as LS means ± standard error. Different letters above bars indicate significant differences between LS means [Number of chickens per group available in Supplementary Table S2].

Finally, when data of all animals were used (both females and males), categorizing breasts according to the presence of a single myopathy or concurrent myopathies (two or three at the same time) to confirm which was the most relevant condition affecting meat quality (Figure 4), significant differences were recorded for final pH, cooking losses, and shear force. As for pH, a lower value was recorded in normal breasts compared to those exhibiting WB alone or in combination with WS (5.90 vs 5.94; *p* < 0.05), while other defective meat showed intermediate values. As for cooking losses, lower values were recorded in normal and WS breasts (23.6% and 25.0%) compared to breasts exhibiting only WB (29.4%) or WB combined with WS (26.5%) and WS combined with SM (27.7%), or breasts exhibiting the three myopathies at the same time (29.5%), while intermediate losses were recorded in breasts affected by SM alone (*p* < 0.001). Finally, shear force was lower in normal meat compared to combined WS and WB breast (2.92 kg/g vs 3.41 kg/g) with the highest values in WB meat (4.30 kg/g) compared to all other types of meat (*p* < 0.001).

## 4 Discussion

This study reviewed the rate of occurrence of different myopathies and was intended to clarify the role of some traits that can affect their occurrence based on data collected per single chicken under controlled conditions, besides to identify recurrent variations of meat quality according to the different myopathies.

Overall, the present study found the highest occurrence for WS myopathy (on average 64.7%) compared to WB and SM, which is consistent with literature data. Nevertheless, large differences among findings exist based on the ontogenetic and environmental factors tested in the different conditions. Kuttappan et al. (2017a) reported a high incidence of mild WS in 6-week-old broilers (78.4%), while the incidence of severe or very severe WS was higher at 9 weeks compared to 6 weeks (69% vs 14%), stipulating the adverse effect of older age and higher weight on the occurrence of severe myopathies. In an earlier study, Kuttappan et al. (2013) reported an incidence of 55.8% of breasts showing moderate and severe WS when birds were slaughtered between 59 and 63 days of age, whereas Petracci et al. (2013) observed only 12.0% of WS occurrence in birds with lower live weight, slaughtered earlier (45–54 days). Reports by Lorenzi et al. (2014) described a prevalence of 43.1% of WS fillets which reached 60.3% in broilers with higher slaughter weight. More recent reports (Petracci et al., 2019; Che et al., 2022b) showed high WS occurrence (>90%) which could be partly attributed to increased awareness of the issue and invested efforts to monitor and better understand the etiology and mechanisms leading to myopathies occurrence (Soglia et al., 2021).

Based on the results of the risk factor analysis, breast weight and breast yield significantly influenced WS occurrence whereas the multivariate model did not find a significant effect of the daily growth rate or the slaughter weight on the occurrence of this myopathy. Overall, our results corroborate previous studies (Alnahhas et al., 2016; Aguirre et al., 2020) reporting the association between WS and fillet weight. Kuttappan et al. (2012a) already observed an association of increased fillet weight and yield with the WS severity degree. Based on the examination of 2,600 fillets from both sexes divided into different fillet-weight groups, Bowker et al. (2019) reported that WS scores slightly increased as fillet weight increased above 450 g. Our multivariate logistic regression model using data of both sexes also showed a significant effect of breast weight and yield on WS. In fact, in both sexes, the degree of the breast development as such and respect to the whole body is likely the challenging factor for the occurrence of white striping, whereas breast yields are rather similar in the two sexes as it was found in previous studies (Hussein et al., 2019; Santos et al., 2021).

As for the other myopathies, relating flock data of growth to individual data of myopathy, Che et al. (2022b) recently found that increased live weight is a risk factor for both WB and SM occurrence. Stated the high correlation between slaughter weight and daily weight gain in our data set (*R* ^2^: 0.86; data not reported in tables), this result is consistent with our observations. In fact, males with high daily weight gain had significantly higher odds of WB occurrence compared to those with low and intermediate daily weight gain. We also found that females with high slaughter weight had significantly higher odds of SM occurrence.

Our analyses clearly identified sex as a risk factor for WB or SM. On average, WB occurrence was 25.2% in males and 12.1% in females, while an opposite behaviour was observed for SM averaging at 36.7% in females and 3.48% in males. Consistently, Santos et al. (2021) found a higher WB occurrence in males than females (41.9% vs 26.8%); Caldas-Cueva et al. (2021) found an approximately three times higher occurrence of moderate to severe WB myopathy in males compared to females; and Lake et al. (2021) reported a higher prevalence of WB (87% vs 71%) and WS (87% vs 73%) in males than females. As for SM, the only available data by Che et al. (2022b) report an average occurrence of 36.3% on a sample size of 9,250 chickens (average live weight at slaughter: 2.36 kg) collected in two slaughterhouses in Ontario (Canada) from flocks comprising only males, only females, and both sexes, whereas they did not identify sex as a risk factor.

Thus, based on the above results and available literature, we could argue that WS is likely associated to the general suffering of muscle fibers in the heaviest breasts in which muscle hypertrophy compromises the normal fiber metabolism regardless from sex. On the other hand, daily growth rate and slaughter weight have been identified as risk factors for WB and SM with higher odds of showing the myopathies in chickens with the highest growth performance. Possibly the different susceptibility to WB and SM of males and females depends on their different growth and metabolism (Kuttappan et al., 2013; Lee et al., 2017; Cui et al., 2021). Indeed, growth differences between sexes are the result of different hormones that influence metabolic processes (Varlamov et al., 2015), besides the impact of gut microbiota (Lee et al., 2017; Cui et al., 2021), that in turn can be predisposing factors for one or another myopathy. Cui et al. (2021) reported that the proteins involved in the process of transporting glucose and affecting the utilization rate of glycogen were higher in males than in female broilers, which would consequently impact body weight. Differences observed at the breast level suggested that increased expression of fat metabolism, oxidative stress response, antiangiogenesis, and connective tissue proliferation genes make male broilers more susceptible to WB compared to females (Brothers et al., 2019). Glycan biosynthesis and metabolism, lipid metabolism, and carbohydrate metabolism have been profiled in male broiler chickens as discriminating body weight groups (Lee et al., 2017). Finally, profiling of predictive metagenome functions from bacterial communities revealed that fructose, mannose, and galactose metabolisms (carbohydrate metabolism) and arachidonic acid metabolism (lipid metabolism) may be related to male broiler chickens, whereas progesterone-mediated oocyte (endocrine system), methane metabolism (energy metabolism), peptidoglycan biosynthesis (glycan biosynthesis and metabolism), glycerophospholipid metabolism, and lipid biosynthesis proteins (lipid metabolism) may be related to female broiler chickens (Lee et al., 2017). Nevertheless, relationships between metabolic patterns in the two sexes and myopathies susceptibility require further investigations especially for SM.

Conversely, as for relationships between meat quality and metabolic changes in muscle affected by myopathies, more information is already available even if differences in meat quality between normal and defective meat are not always consistent across studies. Based on our dataset, the myopathy that mostly affected meat quality was WB, with major changes for technological and sensorial traits, such as pH, cooking losses and shear force.

As for pH, some previous findings reported higher pH also in meat with WS (Mudalal et al., 2015), besides WB (Bowker and Zhuang, 2016; Baldi et al., 2020). Indeed, the severity of the myopathy can influence meat pH: greater values were previously found only in breasts affected with severe WS (Petracci et al., 2013) or severe WB (Campo et al., 2020). Moreover, consistently with our results, some studies showed a greater increase in pH value when WS and WB were simultaneously present (Tasoniero et al., 2016; Zambonelli et al., 2016; Kuttappan et al., 2017a). Finally, the pH of meat affected by SM has been found to be either higher (Baldi et al., 2018) or similar (Campo et al., 2020; Pascual et al., 2020b) to normal meat.

The greater ultimate pH of breasts affected by myopathies has been attributed to a reduced glycolytic potential (Berri et al., 2001; Berri et al., 2007) due to reduced carbohydrate metabolism (Kuttappan et al., 2017a, b). Proteomic analysis, comparing normal breasts and those with severe myopathy, revealed that in affected breast muscles there was a down-regulation of carbohydrate metabolic pathways related to reduced glycolysis, gluconeogenesis, tricarboxylic acid cycle, glycogen degradation, and pyruvate fermentation to lactate (Kuttappan et al., 2017b). Indeed, in heavier breast muscles, a lower storage of glycogen has been reported which has been related to their higher final pH (Le Bihan-Duval et al., 2008). In addition, the greater pH of WB samples have been attributed to lower buffering capacity coupled with reduced glycolytic potential resulting in decreased H+ accumulation (Baldi et al., 2020).

As for cooking losses, the compromised structural integrity of the muscle tissues compared to normal meat likely accounted for the higher values found in WB meat in our analyses. In particular, fiber degeneration and decreased myofibrillar proteins (Mudalal et al., 2015; Soglia et al., 2016; Tijare et al., 2016) can lead to greater mobility of water within the meat structure. In details, a great increase in proportion and mobility of extra-myofibrillar water fraction and a greater mobility of intra-myofibrillar water has been measured in WB and WS/WB meat compared to normal meat (Soglia et al., 2016). The relative abundance of the extra-myofibrillar water and the mobility of intra-myofibrillar water affect drip losses of WB meat, while the mobility of intra-myofibrillar water is crucial for cooking losses (Pang et al., 2020). Indeed, compared to normal meat, higher dripping and cooking losses have been reported also in SM meat (Soglia et al., 2016; Baldi et al., 2018; Pascual et al., 2021).

As for texture and tenderness, among the most important indicators of meat quality (Warner et al., 2022), results about changes due to myopathies are often inconsistent among studies, also as a consequence of differences in meat status (fresh vs frozen), cooking process (raw vs cooked), severity degree of myopathies, sampled cuts, textural methods and, within the same method, measurement conditions (Soglia et al., 2017; Baldi et al., 2019; Pascual et al., 2021). For example, at low compression rates, the resistance is mainly due to the myofiber since the connective tissue has the property of expanding due to its elasticity without interfering with the muscle resistance, whereas at higher compression rates the connective tissue is an important part in the resistance of the muscle (Campo et al., 2000; Campo et al., 2020). Several authors (Mudalal et al., 2015; Dalgaard et al., 2018; Maxwell et al., 2018; Xing et al., 2020) did not find differences in texture between normal, WB, and WS + WB fillets. Other authors reported that differences between normal and WB meat found in raw samples disappeared after applying a thermal process (Soglia et al., 2017; Campo et al., 2020). On the other hand, in agreement with previous results (Tasoniero et al., 2016), across our dataset the highest force was required to cut cooked WB meat which has been attributed to the higher collagen content of WB fillets (Baldi et al., 2019).

Differently, regarding WS, since fat deposition tends to be higher in this meat (Velleman et al., 2010; Mudalal et al., 2015; Radaelli et al., 2017; Soglia et al., 2017), a comparable tenderness between WS and normal meat could be expected also as a results of the disruption of connective tissue cross-linkages in WS muscle fibers (Nishimura, 2010). On the other hand, in SM meat, reduced tenderness is attributed to the reduced collagen cross-linking degree of defective breasts (Baldi et al., 2019, 2021). In fact, Baldi et al. (2019) did not find differences in the texture between raw SM and normal samples, while Pascual et al. (2021) found that only the Meullenet–Owens razor blade test could distinguish between cooked normal and SM fillets.

Consistently with the present results, other studies reported that myopathies did not influence colour indices of breast meat (Zambonelli et al., 2016; Brambila et al., 2017). Nevertheless, within a single trial, Pascual et al. (2020b) found that SM and WB fillets had significantly higher lightness values than normal ones, while Petracci et al. (2013) and Tasoniero et al. (2016) did not find a difference in lightness between normal and defective meat. On the other hand, a higher yellowness (Mudalal et al., 2015) was found in defective compared to normal meat. Indeed, Campo et al. (2020) highlighted the effect of the severity degree where severe WB decreased lightness and increased redness and yellowness. The presence of WS resulted in redder meat, while more severe WS breasts showed higher lightness and yellowness; a moderate SM only increased yellowness (Pascual et al., 2021). Kuttappan et al. (2017a) also reported increased yellowness of fillets affected by severe WS, WB, and WS/WB compared with normal meat. Petracci et al. (2017) associated changes in colour of defective meat with increased fibrotic responses and reduced haem pigment levels.

Nevertheless, the impact on consumers’ acceptance has been found to be relevant for all myopathies. In fact, raw WS meat resulted in lower acceptability and purchase intent compare to unaffected meat (de Carvalho et al., 2020) and consumer acceptance decreased along with the severity of WS (Kuttappan et al., 2012b). As for WB, neither traditional nor clean label marinades mask the undesirable eating characteristics of this defective meat and changes will be noticeable to consumers (Jarvis et al., 2020). Data about consumers’ perception of SM meat have not been published, but the consumers’ reaction is obvious and easily predictable.

Finally, as for the most relevant effects of sex on meat quality across all trials, the higher meat pH of males compared to females resulted in a higher water retention that is in turn responsible for a higher reflection of light and, thus, for the higher lightness index we measured in males (Campo et al., 2020). The higher pH of males could be related to their higher breast weights. In fact, a negative correlation between breast muscle weight and glycogen stored in the muscle, i.e., a positive correlation of breast weight with the final pH, has been reported (Le Bihan-Duval et al., 2008). While we measured a higher shear force for cutting meat of male chickens, other authors found a lower value than female chickens which was attributed to the smaller fiber diameter in males (Cygan-Szczegielniak and Bogucka, 2021).

In conclusion, among the growth and slaughter traits, our analysis identified breast development, measured as breast weight and breast yield at an individual level, as a potential risk factor for the prevailing myopathy, i.e., white striping. Conversely, a high growth, measured as daily weight gain and slaughter weight, was the risk factor for wooden breast and spaghetti meat. Along with the information available in literature, these results can pave the way for the evaluation of the etiopathogenic mechanisms that can trigger the onset of white striping on one side and wooden breasts or spaghetti meat on the other one. Additionally, we also found a different probability of having wooden breasts or spaghetti meat in females compared to males, which still deserves specific investigations. Finally, based on the results of the present analyses, only WB meat was clearly different for some technological and sensorial properties, which can play a role also in meat processing.

## Data Availability

The original contributions presented in the study are included in the article/Supplementary Material, further inquiries can be directed to the corresponding author.
