# Supplementary material for: Factors Affecting Breast Myopathies in Broiler Chickens and Quality of Defective Meat: A Meta-Analysis
Source: Front Physiol. 2022 Jul 1;13:933235. doi: 10.3389/fphys.2022.933235 (PMC9283645; doi:10.3389/fphys.2022.933235)
Supplement: Supplementary file 1 [file Table1.docx]

**Table S1.** Number and percentage (between parentheses) of chickens with normal breasts or defective breasts presenting a single myopathy or associated myopathies: whole data set

|  | All chickens | Females | Males |
| --- | --- | --- | --- |
|  | n | n | n |
| Normal | 354 (27.7%) | 156 (28.4%) | 198 (27.2%) |
| Only white striping | 576 (45.1%) | 203 (37.0%) | 373 (51.2%) |
| Only wooden breast | 46 (3.6%) | 20 (3.6%) | 26 (3.6%) |
| Only spaghetti meat | 39 (3.1%) | 39 (7.1%) | 0 (0.0%) |
| White striping and wooden breast | 202 (15.8%) | 82 (14.9%) | 120 (16.5%) |
| White striping and spaghetti meat | 41 (3.2%) | 34 (6.2%) | 7 (1.0%) |
| Wooden breast and spaghetti meat | - | - | - |
| All myopathies | 20 (1.6%) | 15 (2.7%) | 5 (0.7%) |

**Table S2.** Number of chickens with normal breasts or defective breasts presenting a single myopathy or associated myopathies: data set used for the evaluation of the effect of myopathy occurrence on meat quality

|  | All chickens | Females | Males |
| --- | --- | --- | --- |
|  | n | n | n |
| Normal | 236 | 109 | 127 |
| Only white striping | 380 | 143 | 237 |
| Only wooden breast | 30 | 17 | 13 |
| Only spaghetti meat | 33 | 33 | - |
| White striping and wooden breast | 166 | 75 | 91 |
| White striping and spaghetti meat | 32 | 27 | 5 |
| Wooden breast and spaghetti meat | - | - | - |
| All myopathies | 13 | 9 | 4 |

**Table S3**. Univariate analysis results of myopathy occurrence evaluating sex as potential influencing factor.

|  | Myopathy | | P-value | Odds ratio (95%CI) |
| --- | --- | --- | --- | --- |
| *White striping* | WS 0 (n = 439) | WS 1 (n = 839) |  |  |
| Males (Ref) | 223 (30.6%) | 505 (69.4%) | - | - |
| Females | 216 (39.3%) | 334 (60.7%) | <0.01 | 0.68 (0.54 – 0.86) |
|  |  |  |  |  |
| *Wooden breast* | WB 0 (n = 1058) | WB 1 (n = 220) |  |  |
| Males (Ref) | 557 (79.3%) | 151 (20.7%) | - | - |
| Females | 481 (87.5%) | 69 (12.5%) | <0.001 | 0.55 (0.40 – 0.75) |
|  |  |  |  |  |
| *Spaghetti meat* | SM 0 (n = 584) | SM 1 (n = 159) |  |  |
| Males (Ref) | 338 (96.3%) | 13 (3.70%) | - | - |
| Females | 246 (62.8%) | 146 (37.2%) | <0.001 | 15.4 (8.55 – 27.8) |

WS = white striping; WB = wooden breast; SM = spaghetti meat; CI = confidence interval; Ref = reference. Sample size per each sex is presented outside the parenthesis.

**Table S4**. Potential influencing factors of white striping occurrence in female and male broiler chickens.

|  | White striping | | P-value | Odds ratio (95% CI) |
| --- | --- | --- | --- | --- |
|  | WS = 0 | WS = 1 |  |  |
| *Females* |  |  |  |  |
| Genotype |  |  |  |  |
| A (Ref) | 18 (58.1%) | 13 (41.9%) | - | - |
| B | 185 (39.4%) | 285 (60.6%) | 0.86 | 2.13 (1.02 – 4.46) |
| C | 12 (25.0%) | 36 (75.0%) | <0.01 | 4.15 (1.58 – 10.9) |
| Daily weight gain |  |  |  |  |
| Medium: 63.3 – 67.7 g/d (Ref) | 60 (32.8%) | 123 (67.2%) | - | - |
| Low: <63.3 g/d | 92 (50.0%) | 92 (50.0%) | <0.001 | 0.48 (0.32 – 0.75) |
| High: >67.7 g/d | 63 (34.6%) | 119 (65.4%) | 0.14 | 0.92 (0.60 – 1.42) |
| Slaughter weight |  |  |  |  |
| Medium: 2720 – 2935 g (Ref) | 61 (33.3%) | 122 (66.7%) | - | - |
| Low: <2720 g | 87 (47.5%) | 96 (52.5%) | <0.01 | 0.55 (0.36 – 0.84) |
| High: >2935 g | 67 (36.6%) | 116 (63.4%) | 0.41 | 0.87 (0.56 – 1.33) |
| Breast weight, g |  |  |  |  |
| Medium: 798 – 871 g (Ref) | 49 (32.9%) | 100 (67.1%) | - | - |
| Low: <798 g | 84 (54.9%) | 69 (45.1%) | <0.001 | 0.40 (0.25 – 0.64) |
| High: >871 g | 43 (28.5%) | 108 (71.5%) | <0.001 | 1.23 (0.75 – 2.01) |
| Breast yield, % |  |  |  |  |
| Medium: 40.0 – 42.0% (Ref) | 65 (43.1%) | 86 (56.9%) | - | - |
| Low: <40.0% | 67 (44.7%) | 83 (55.3%) | 0.06 | 0.94 (0.59 – 1.48) |
| High: >42.0% | 44 (29.0%) | 108 (71.0%) | <0.01 | 1.86 (1.15 – 2.99) |
|  |  |  |  |  |
| *Males* |  |  |  |  |
| Genotype |  |  |  |  |
| A (Ref) | 14 (43.8%) | 18 (56.2%) | - | - |
| B | 168 (31.1%) | 372 (68.9%) | 0.05 | 1.72 (0.84 – 3.55) |
| C | 41 (26.3%) | 115 (73.7%) | 0.49 | 2.18 (1.00 – 4.78) |
| Daily weight gain |  |  |  |  |
| Medium: 70.7 – 78.0 g/d (Ref) | 72 (29.3%) | 174 (70.7%) | - | - |
| Low: <70.7 g/d | 99 (40.7%) | 144 (59.3%) | <0.001 | 0.60 (0.41 – 0.88) |
| High: >78.0 g/d | 52 (21.8%) | 187 (78.2%) | <0.001 | 1.49 (0.99 – 2.24) |
| Slaughter weight |  |  |  |  |
| Medium: 3225 – 3325 g (Ref) | 73 (30.3%) | 168 (69.7%) | - | - |
| Low: <3225 g | 96 (39.5%) | 147 (60.5%) | <0.001 | 0.67 (0.46 – 0.97) |
| High: >3325 g | 54 (22.1%) | 190 (77.9%) | <0.001 | 1.53 (1.02 – 2.30) |
| Breast weight, g |  |  |  |  |
| Medium: 935 – 1040 g (Ref) | 40 (25.3%) | 118 (74.7%) | - | - |
| Low: <935 g | 63 (39.9%) | 95 (60.1%) | <0.001 | 0.51 (0.32 – 0.83) |
| High: >1040 g | 36 (22.5%) | 124 (77.5%) | <0.05 | 1.17 (0.70 – 1.96) |
| Breast yield, % |  |  |  |  |
| Medium: 38.4 – 40.0% (Ref) | 46 (28.6%) | 115 (71.4%) | - | - |
| Low: <38.4% | 58 (38.7%) | 92 (61.3%) | <0.01 | 0.63 (0.40 – 1.02) |
| High: >40.0% | 35 (21.2%) | 130 (78.8%) | <0.01 | 1.49 (0.90 – 2.47) |

WS = white striping; CI = confidence interval; Ref = reference.

**Table S5**. Potential influencing factors of wooden breast occurrence in female and male broiler chickens.

|  | Wooden breast | | P-value | Odds ratio (95% CI) |
| --- | --- | --- | --- | --- |
|  | WB = 0 | WB = 1 |  |  |
| *Females* |  |  |  |  |
| Genotype |  |  |  |  |
| A (Ref) | 26 (83.9%) | 5 (16.1%) | - | - |
| B | 407 (86.6%) | 63 (13.4%) | 0.13 | 0.11 (0.01 – 1.00) |
| C | 47 (97.2%) | 1 (2.08%) | 0.05 | 0.81 (0.30 – 2.17) |
| Daily weight gain |  |  |  |  |
| Medium: 63.3 – 67.7 g/d (Ref) | 158 (86.4%) | 25 (13.7%) | - | - |
| Low: <63.3 g/d | 163 (88.6%) | 21 (11.4%) | 0.56 | 0.91 (050 – 1.68) |
| High: >67.7 g/d | 159 (87.4%) | 23 (12.6%) | 0.96 | 0.81 (0.44 – 1.51) |
| Slaughter weight |  |  |  |  |
| Medium: 2720 – 2935 g (Ref) | 155 (84.7%) | 28 (15.3%) | - | - |
| Low: < 2720 g | 163 (89.1%) | 20 (10.9%) | 0.44 | 0.68 (0.37 – 1.26) |
| High: >2936 g | 162 (88.55) | 21 (11.5%) | 0.62 | 0.72 (0.39 – 1.32) |
| Breast weight, g |  |  |  |  |
| Medium: 798 – 871 g (Ref) | 128 (85.9%) | 21 (14.1%) |  |  |
| Low: <798 g | 138 (90.2%) | 15 (9.80%) | 0.23 | 0.66 (0.33 – 1.34) |
| High: >872 g | 127 (84.1%) | 24 (15.9%) | 0.13 | 1.15 (0.61 – 2.17) |
| Breast yield, % |  |  |  |  |
| Medium: 40.0 – 42.0% (Ref) | 133 (88.1%) | 18 (11.9%) | - | - |
| Low: <40.0% | 133 (88.7%) | 17 (11.3%) | 0.43 | 0.94 (0.47 – 1.91) |
| High: >42.0% | 127 (83.6%) | 25 (16.4%) | 0.15 | 1.46 (0.76 – 2.80) |
|  |  |  |  |  |
| *Males* |  |  |  |  |
| Genotype |  |  |  |  |
| A (Ref) | 28 (87.5%) | 4 (12.5%) | - | - |
| B | 402 (74.4%) | 138 (25.6%) | <0.001 | 2.40 (0.83 – 6.97) |
| C | 147 (94.2%) | 9 (5.77%) | <0.01 | 0.43 (0.12 – 1.49) |
| Daily weight gain |  |  |  |  |
| Medium: 70.7 – 78.0 g/d (Ref) | 204 (82.9%) | 42 (17.1%) | - | - |
| Low: <70.7 g/d | 203 (83.5%) | 40 (16.5%) | 0.06 | 0.96 (0.60 – 1.54) |
| High: >78.0 g/d | 170 (71.1%) | 69 (28.9%) | <0.001 | 1.97 (1.28 – 3.04) |
| Slaughter weight |  |  |  |  |
| Medium: 3225 – 3325 g (Ref) | 192 (79.7%) | 49 (20.3%) | - | - |
| Low: <3225 g | 195 (80.3%) | 48 (19.8%) | 0.65 | 0.97 (0.62 – 1.51) |
| High: >3325 g | 190 (77.9%) | 54 (22.1%) | 0.51 | 1.14 (0.72 – 1.72) |
| Breast weight, g |  |  |  |  |
| Medium: 935 – 1040 g (Ref) | 114 (72.2%) | 44 (27.9%) | - | - |
| Low: <935 g | 128 (81.0%) | 30 (19.0%) | 0.19 | 0.61 (0.36 – 1.03) |
| High: >1040 g | 126 (78.8%) | 34 (21.3%) | 0.65 | 0.70 (0.42 – 1.17) |
| Breast yield, % |  |  |  |  |
| Medium: 38.4 – 40.0% (Ref) | 126 (78.3%) | 35 (21.7%) | - | - |
| Low: <38.4% | 122 (81.3%) | 28 (18.7%) | 0.17 | 0.83 (0.47 – 1.44) |
| High: >40.0% | 120 (72.7%) | 45 (27.3%) | 0.08 | 1.35 (0.81 – 2.24) |

WB = wooden breast; CI = confidence interval; Ref = reference.

**Table S6**. Potential influencing factors of spaghetti meat occurrence in female and male broiler chickens.

|  | Spaghetti meat | | P-value | Odds ratio (95% CI) |
| --- | --- | --- | --- | --- |
|  | SM = 0 | SM = 1 |  |  |
| *Females* |  |  |  |  |
| Genotype |  |  |  |  |
| B | 246 (62.8%) | 146 (37.2%) | - | - |
| Daily weight gain |  |  |  |  |
| Medium: 63.3 – 67.7 g/d (Ref) | 87 (59.6%) | 59 (40.4%) | - | - |
| Low: <63.3 g/d | 94 (71.2%) | 38 (28.8%) | <0.05 | 0.57 (0.36 – 0.98) |
| High: >67.7 g/d | 65 (57.0%) | 49 (43.0%) | 0.11 | 1.12 (0.68 – 1.83) |
| Slaughter weight |  |  |  |  |
| Medium: 2720 – 2935 g (Ref) | 96 (65.3%) | 51 (34.7%) | - | - |
| Low: <2720 g | 96 (66.7%) | 48 (33.3%) | 0.16 | 0.94 (0.58 – 1.53) |
| High: >2936 g | 54 (53.5%) | 47 (46.5%) | <0.05 | 1.64 (0.98 – 2.75) |
| Breast weight, g |  |  |  |  |
| Medium: 798 – 871 g (Ref) | 67 (58.8%) | 47 (41.2%) | - | - |
| Low: <798 g | 81 (67.5%) | 39 (32.5%) | <0.05 | 0.69 (0.40 – 1.17) |
| High: >872 g | 57 (51.8%) | 53 (48.2%) | <0.05 | 1.33 (0.78 – 2.25) |
| Breast yield, % |  |  |  |  |
| Medium: 40.0 – 42.0% (Ref) | 69 (59.0%) | 48 (41.0%) | - | - |
| Low: <40.0% | 66 (59.5%) | 45 (40.5%) | 0.84 | 0.95 (0.56 – 1.60) |
| High: >42.0% | 70 (60.3%) | 46 (39.7%) | 0.97 | 0.98 (0.58 – 1.66) |
|  |  |  |  |  |
| *Males* |  |  |  |  |
| Genotype |  |  |  |  |
| B | 338 (96.3%) | 13 (3.70%) | - | - |
| Daily weight gain |  |  |  |  |
| Medium: 70.7 – 78.0 g/d (Ref) | 99 (94.3%) | 6 (5.71%) | - | - |
| Low: <70.7 g/d | 99 (96.1%) | 4 (3.88%) | 0.21 | 0.67 (0.18 – 2.44) |
| High: >78.0 g/d | 140 (97.9%) | 3 (2.10%) | 0.86 | 0.35 (0.09 – 1.45) |
| Slaughter weight |  |  |  |  |
| Medium: 3225 – 3325 g (Ref) | 115 (96.6%) | 4 (3.36%) | - | - |
| Low: <3225 g | 123 (95.4%) | 6 (4.65%) | 0.52 | 1.40 (0.39 – 5.10) |
| High: >3325 g | 100 (97.1%) | 3 (2.91%) | 0.64 | 0.86 (0.19 – 3.95) |
| Breast weight, g |  |  |  |  |
| Medium: 935 – 1040 g (Ref) | 83 (95.4%) | 4 (4.60%) | - | - |
| Low: <935 g | 106 (96.4%) | 4 (3.64%) | 0.73 | 0.78 (0.19 – 3.22) |
| High: >1040 g | 57 (98.3%) | 1 (1.72%) | 0.41 | 0.36 (0.04 – 3.34) |
| Breast yield, % |  |  |  |  |
| Medium: 38.4 – 40.0% (Ref) | 86 (96.6%) | 3 (3.37%) | - | - |
| Low: <38.4% | 85 (100%) | 0 (0.00%) | 0.94 | - |
| High: >40.0% | 75 (92.6%) | 6 (7.41%) | 0.93 | 2.29 (0.55 – 9.49) |

SM = spaghetti meat; CI = confidence interval; Ref = reference.

**Table S7**. Factors influencing white striping occurrence in broiler chickens and extracted by forward selection in the multivariate logistic regression analysis (n = 929)

| Variable | Estimate | SE | Odds ratio | 95% CI | | *P value* |
| --- | --- | --- | --- | --- | --- | --- |
|  |  |  |  | Lower | Upper |  |
| *Genotype B* |  |  |  |  |  |  |
| Intercept | 0.72 | 0.08 |  |  |  | <0.001 |
| Breast weight |  |  |  |  |  |  |
| Medium: 850 – 960 g (Ref) | - | - | - | - | - | - |
| Low: <850 g | -0.53 | 0.11 | 0.53 | 0.37 | 0.75 | <0.001 |
| High: >960 g | 0.42 | 0.12 | 1.37 | 0.91 | 2.05 | <0.001 |
| *Genotype C* |  |  |  |  |  |  |
| Intercept | 0.73 | 0.30 |  |  |  | <0.05 |
| Breast weight |  |  |  |  |  |  |
| Medium: 850 – 960 g (Ref) | - | - | - | - | - | - |
| Low: <850 g | -1.10 | 0.51 | 0.28 | 0.05 | 1.48 | <0.05 |
| High: >960 g | 0.92 | 0.37 | 2.08 | 0.64 | 6.73 | <0.05 |
| Breast yield |  |  |  |  |  |  |
| Medium: 39.0 – 40.7% (Ref) | - | - | - | - | - | - |
| Low: <39.0% g | -1.10 | 0.38 | 0.30 | 0.08 | 1.10 | <0.001 |
| High: >40.7% | 1.00 | 0.40 | 2.48 | 0.34 | 9.67 | <0.05 |

SE = standard error; CI = Confidence interval; Ref = reference; genotype A: Ross 708; genotype B: Ross 308; genotype C: Cobb 500

**Table S8**. Factors influencing wooden breast occurrence in male broiler chickens of three different genotypes and extracted by forward selection in the multivariate logistic regression analysis (n = 728).

| Variable | Estimate | SE | Odds ratio | 95% CI | | *P value* |
| --- | --- | --- | --- | --- | --- | --- |
|  |  |  |  | Lower | Upper |  |
| *Genotype B* |  |  |  |  |  |  |
| Intercept | -0.83 | 0.13 |  |  |  | <0.001 |
| Breast yield |  |  |  |  |  |  |
| Medium: 38.4 – 40.0% (Ref) | - | - | - | - | - | - |
| Low: <38.4% | -0.41 | 0.16 | 0.72 | 0.43 | 1.22 | <0.05 |
| High: >40.0% | 0.50 | 0.20 | 1.79 | 0.93 | 3.42 | <0.05 |

SE = standard error; CI = Confidence interval; Ref = reference; genotype A: Ross 708; genotype B: Ross 308; genotype C: Cobb 500

**Table S9**. Factors influencing spaghetti meat occurrence in female broiler chickens of three different genotypes and extracted by forward selection in the multivariate logistic regression analysis (n = 344)

| Variable | Estimate | SE | Odds ratio | 95% CI | | *P value* |
| --- | --- | --- | --- | --- | --- | --- |
|  |  |  |  | Lower | Upper |  |
| *Genotype A* |  |  |  |  |  |  |
| Intercept | -0.30 | 0.11 |  |  |  | <0.05 |
| Slaughter weight |  |  |  |  |  |  |
| Medium: 2720 – 2935 g (Ref) | - | - | - | - | - | - |
| Low: < 2720 g | -0.34 | 0.16 | 0.87 | 0.53 | 1.42 | <0.05 |
| High: >2936 g | 0.54 | 0.18 | 2.10 | 1.18 | 3.76 | <0.01 |
| *Genotype B* |  |  |  |  |  |  |
| Intercept | -0.30 | 0.12 |  |  |  | <0.05 |
| Slaughter weight |  |  |  |  |  |  |
| Medium: 2720 – 2935 g (Ref) | - | - | - | - | - | - |
| Low: < 2720 g | -0.34 | 0.16 | 0.87 | 0.53 | 1.42 | <0.05 |
| High: >2936 g | 0.54 | 0.18 | 2.10 | 1.18 | 3.76 | <0.01 |

SE = standard error; CI = Confidence interval; Ref = reference; genotype A: Ross 708; genotype B: Ross 308; genotype C: Cobb 500.
